# Early evidence of anti-PD-1 activity in enzalutamide-resistant prostate cancer

**DOI:** 10.18632/oncotarget.10547

**Published:** 2016-07-12

**Authors:** Julie N. Graff, Joshi J. Alumkal, Charles G. Drake, George V. Thomas, William L. Redmond, Mohammad Farhad, Jeremy P. Cetnar, Frederick S. Ey, Raymond C. Bergan, Rachel Slottke, Tomasz M. Beer

**Affiliations:** ^1^ Division of Hematology/Oncology, Knight Cancer Institute, Oregon Health & Science University, Portland, OR, USA; ^2^ VA Portland Health Care System, Portland, OR, USA; ^3^ Sidney Kimmel Comprehensive Cancer Center and the Brady Urological Institute, Johns Hopkins University School of Medicine, Baltimore, MD, USA; ^4^ Pathology and Laboratory Medicine, Oregon Health and Science University, Portland, OR, USA; ^5^ Robert W. Franz Cancer Research Center, Earle A. Chiles Research Institute, Providence Portland Medical Center, Portland, OR, USA; ^6^ Cell, Developmental, and Cancer Biology Department, Oregon Health and Science University, Portland, OR, USA

**Keywords:** prostate cancer, PD-1, immunotherapy, enzalutamide, Immunology and Microbiology Section, Immune response, Immunity

## Abstract

While programmed cell death 1 (PD-1) inhibitors have shown clear anti-tumor efficacy in several solid tumors, prior results in men with metastatic castration resistant prostate cancer (mCRPC) showed no evidence of activity. Here we report unexpected antitumor activity seen in mCRPC patients treated with the anti-PD-1 antibody pembrolizumab. Patients with evidence of progression on enzalutamide were treated with pembrolizumab 200 mg IV every 3 weeks for 4 doses; pembrolizumab was added to standard dose enzalutamide. Three of the first ten patients enrolled in this ongoing phase II trial experienced rapid prostate specific antigen (PSA) reductions to ≤ 0.2 ng/ml. Two of these three patients had measurable disease upon study entry; both achieved a partial response. There were three patients with significant immune-related adverse events. One had grade 2 myositis, one had grade 3 hypothyroidism, and one had grade 2 hypothyroidism. None of these patients had a response. Two of the three responders had a baseline tumor biopsy. Immunohistochemistry from those biopsies showed the presence of CD3^+^, CD8^+^, and CD163^+^ leukocyte infiltrates and PD-L1 expression. Genetic analysis of the two responders revealed markers of microsatellite instability in one. The surprising and robust responses seen in this study should lead to re-examination of PD-1 inhibition in prostate cancer.

## INTRODUCTION

In 2012, a phase I study of PD-1 inhibition with nivolumab in cancer patients showed activity in melanoma, renal cell and non-small cell lung cancer [1]. Seventeen patients with castration-resistant prostate cancer (CRPC) were included in the study, but there were no objective responses in that group. Since that study, PD-1 inhibition has demonstrated improved survival in phase III studies for non-small cell lung cancer [2, 3], renal cell carcinoma [4], melanoma [5, 6] and bladder cancer [7]. However, given the initial findings in the phase I study and the paucity of PD-L1 staining of previously reported in prostate cancer tissue [8], as well as two negative studies of ipilimumab in CRPC [9, 10], there has been little interest in the examination of this class of immunotherapies in prostate cancer.

We previously reported on two CRPC patients who were exceptional responders to immunotherapy and hypothesized that the immune modulatory actions of androgen receptor blockers may enhance immunotherapy [11-17]. We therefore developed a phase II study to evaluate the efficacy of immuno-hormonal therapy with enzalutamide and the PD-1 inhibitor pembrolizumab in men with metastatic CRPC. Early results from the first 10 patients enrolled on this study are reported here.

## RESULTS

The first ten subjects were enrolled from March 2015 to January 2016. Baseline characteristics are presented in Table 1. As of May 19, 2016, the median number of cycles was 4 (range 2-8). The median follow up was 30 weeks (range 16-55). One patient received pembrolizumab retreatment.

**Figure 1:**
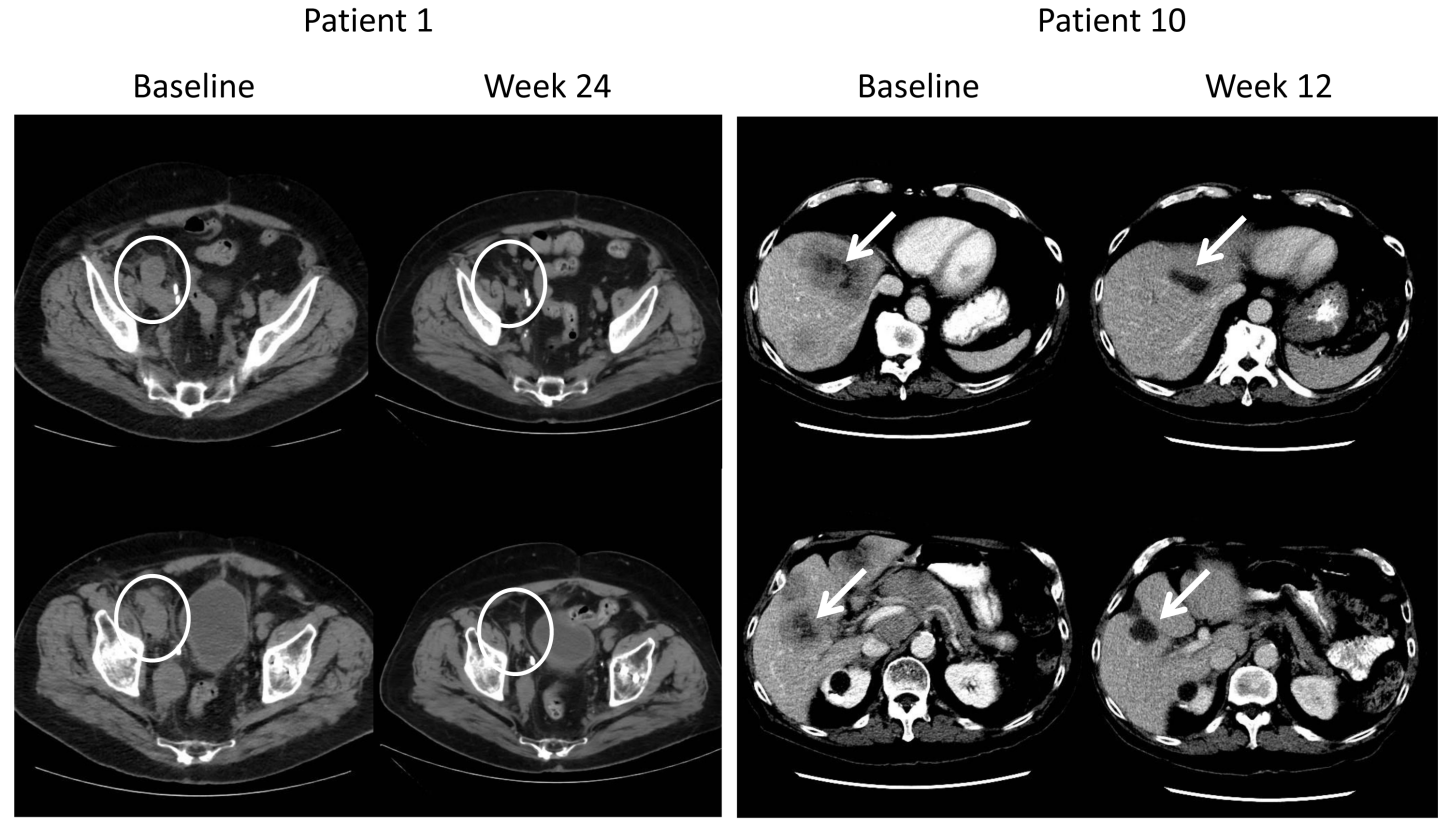
Radiographic Responses in Patients With Measurable Disease

Of the ten patients enrolled, three demonstrated significant antitumor activity (Table 2). Starting from serum PSA of 46, 71, and 2,503 ng/ml, these three patients had a near complete PSA response, reaching a serum PSA of ≤ 0.1 ng/ml. Two of these three patients had measurable soft tissue disease and both had a partial response (Figure 1) with one of these patients experiencing a response in liver metastases. Two of the three responders discontinued opiate analgesics and reported resolution of cancer related pain. These three patients remain free of progression at 30, 55, and 16 weeks of follow-up. Of the remaining 7 patients, three had stable disease of 30, 47 and 50 weeks, which are ongoing, while the remaining 4 patients did not have evidence of clinical benefit. One of the patients without benefit died of prostate cancer.

**Table:** Patient Characteristics

Characteristic	Number of Patients
Patients enrolled	10
Age, years Median Range	72 61-80
Race Caucasian	10
ECOG PS 0 1	3 7
Treatment of the primary Radical prostatectomy Radiation therapy None	6 1 3
Gleason sum at diagnosis ≤6 7 ≥8	1 5 4
Clinical stage at diagnosis (*n* = 4) T1c T2b T2c M1 Pathological stage (*n* = 6) T2 T3 N0 N1	1 1 2 2 3 3 3 3
Sites of metastatic disease Bone only Lymph nodes only Liver and bone Number with measurable disease	7 2 1 3
Lesion that could be biopsied	3
PSA, ng/ml Median Range	25.86 4.13-2502.75
Hemoglobin, g/dl Median Range	12.75 10.3-15.1
Alkaline phosphatase, U/L Median Range	76.5 31-568
Prior therapies Docetaxel for castration sensitive disease Abiraterone Enzalutamide Sipuleucel-T	1 5 10 1
Number of weeks on enzalutamide prior to study Median Range	52 29-230
Using narcotics at baseline	6

ECOG PS – Eastern Cooperative Oncology Group Performance Status

**Table:** Responding Patients*

Patient number	Date of cycle 1	PSA (ng/ml) baseline to nadir	Measurable Disease at Baseline	Best Radiologic Response	MSI	Prior Treatment for mCRPC
1	April 2015	70.65 → 0.08	Yes	PR	present	abi, enz
7	October 2015	46.09 → 0.02	No	N/A	n/a	abi, enz
10	January 2016	2502.75 → < 0.01	Yes	PR	absent	enz

* All responding patients remain on study. PR – partial response; N/A – not applicable (i.e. no baseline biopsy done); MSI – microsatellite instability; abi – abiraterone; enz – enzalutamide

Expected immune toxicities were observed (Table 3). One patient had grade 2 myositis, which resolved with a steroid taper, and pembrolizumab was permanently discontinued. One patient experienced immune-mediated grade 3 hypothyroidism and permanently stopped study treatment. A third patient developed hypothyroidism of uncertain etiology after 2 doses of pembrolizumab. Other adverse events (Table 3) were not judged to be treatment related. Notably, there was no correlation between anti-tumor activity and immune related adverse events. None of the responders had an immune related adverse event.

**Figure 2:**
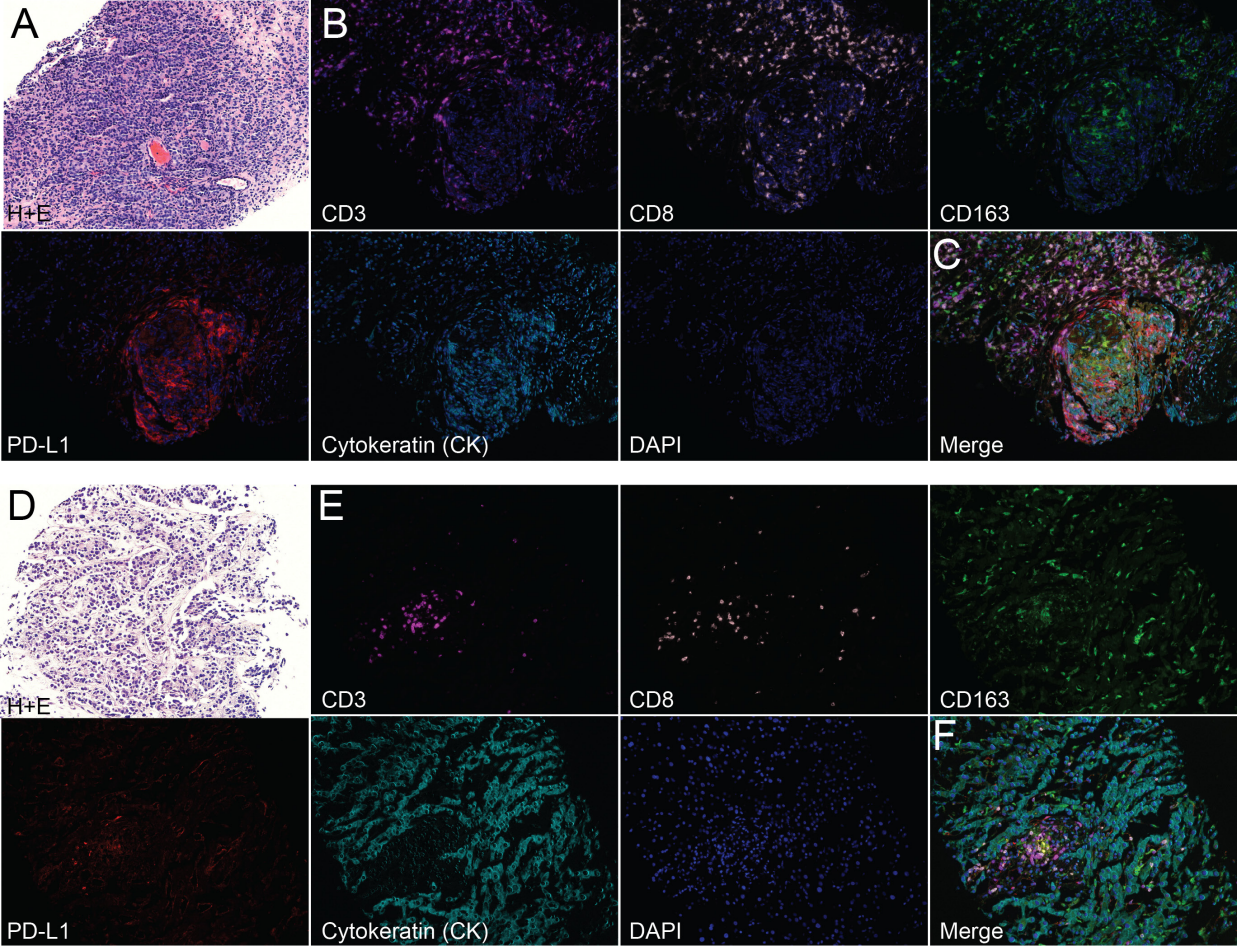
Multi-spectral imaging reveals leukocyte infiltration in biopsies from men with metastatic castrate-resistant prostate cancer (mCRPC) **A**-**C**) Lymph node (LN) and **D**-**F**) liver biopsies were obtained from men with mCRPC. A) H+E and B) single-color images (plus nuclear stain; DAPI) of CD3, CD8, CD163, PD-L1, cytokeratin (CK), DAPI and C) merged image from a LN biopsy of patient A. D) H+E, E) single-color, and F) merged from a liver biopsy of patient B. Note: images depicted in (B-C and E-F) were selected from representative “hot spots” of leukocyte infiltrates in each biopsy. A, D) H+E images 20X; B, C, E, F) multi-spectral images 200X.

Correlative studies: Two of the three responders had a baseline tissue biopsy (i.e. prior to pembrolizumab), and we evaluated the extent of leukocyte infiltration in these biopsies, which were of a lymph node and liver (Figure 2). We detected CD3^+^CD8^+^ T cell and CD163^+^ macrophage infiltrates along with PD-L1 expression in both patients. However, the extent of leukocyte infiltrates and PD-L1 expression were variable. For example, in the lymph node biopsy, pronounced T cell and macrophage infiltrates were detected in and around the tumor. We also observed marked PD-L1 staining that appeared to be expressed on CK^+^ cells. While we also detected pronounced leukocyte infiltration in the liver biopsy, the PD-L1 expression appeared to be associated more closely with infiltrating leukocytes rather than CK^+^ cells. Taken together, these data provide evidence of pre-existing leukocyte infiltration in the liver biopsy in one of the responders. The lymph node biopsy is more difficult to interpret since these cells would be expected to reside there, but the staining for lymphocytes was robust in the tumor deposit itself. Genetic analysis revealed microsatellite instability (MSI) in the baseline biopsy of one of the two responders. Neither of the two non-responders had MSI.

**Table:** Adverse Events

Adverse Event	Grade (Number of subjects)
Cardiac: tachycardic	1 (1)
Ear and labyrinth: vertigo	1 (2)
Gastrointestinal disorders: Abdominal pain Chelitis Constipation Diarrhea Nausea Dysphagia Mucositis	2 (1) 1 (1) 2 (1) 1 (2), 2 (1), 3 (1) 1 (2) 2 (1) 1 (1)
General disorders: Fatigue	2 (1)
Infections: Urinary tract infections	2 (1)
Injury: Fall Fracture	1 (1) 3 (1)
Investigations: ALT increased AST increased CPK increase Weight loss	1 (1), 2 (1) 2 (2) 4 (1) 2 (1)
Metabolism and nutrition disorders: Anorexia	2 (2)
Musculoskeletal and connective tissue disorders: Arthralgias Bone pain Muscle weakness Myalgias Pain	2 (1) 2 (1) 1 (1) 1 (1), 2 (1) 1 (3)
Nervous System Disorders: Confusion Insomnia Myelitis Peripheral sensory neuropathy	1 (1) 1 (1) 3 (1) 1 (1)
Reproductive system disorders: Genital edema	2 (1)
Respiratory, thoracic and mediastinal disorders: Dyspnea	1 (2), 2 (1)
Skin and subcutaneous tissue disorders: maculopapular rash	1 (2)
Vascular disorders: hot flashes	1 (1)
Immune Related Adverse Event (patient) and clinical manifestations	Outcome
Myositis, grade 2 (4) as evidenced by weakness, pain, dysphagia, and grade 4 CPK elevation.	High dose steroid taper one time. Resolved, and pembrolizumab discontinued.
Hypothyroidism, grade 3 (6) presenting with weakness in his limbs and pain in his hands.	Thyroid replacement and high dose steroid taper three times, as the symptoms quickly returned after taper. Currently on third taper with improvement of symptoms. Pembrolizumab discontinued.
Hypothyroidism, grade 2 (8) found on labs.	Thyroid replacement given.

## DISCUSSION

These data show that PSA and objective clinical responses to PD-1 blockade occur in prostate cancer patients progressing on enzalutamide. These results stand in contrast with those observed in the initial phase I experience with nivolumab. There are several possible explanations for this possible discrepancy.

One explanation for this discrepancy could be the agents employed, i.e. our study used the humanized anti-PD-1 antibody pembrolizumab whereas earlier work used the fully human IgG4 antibody nivolumab. Since responses to these two agents have generally been quite comparable in other cancers, i.e. melanoma and NSCLC, this seems unlikely, however at this stage we cannot exclude the possibility that some heretofore unanticipated differences between these two agents that are relevant to prostate cancer exist [2, 3, 5, 6, 18].

A second possibility involves the disease state, in the prior phase I study patients could have received up to five prior treatments for mCRPC, whereas here patients could not have had prior chemotherapy for mCRPC and thus might have had fewer previous treatments and an ECOG status ≤ 1.

A third possibility concerns the concept of mutation burden particularly due to mismatch repair (MMR) defects and/or microsatellite instability. Recent studies in CRC and other tumor types show remarkable efficacy for PD-1 blockade in such patients. A hypermutated genotype related to MMR repair defects has been reported in mCRPC patients [19]. Microsatellite instability (MSI) was thought to be rare in prostate cancer (< 2%), but one analysis found that 7 of 60 men with advanced prostate cancer had MSI[19,20]. Indeed, in the two responders with baseline biopsies, we found evidence of microsatellite instability in one but not the other. Additional work is needed to determine the relationship between MSI and response in prostate cancer.

Although results are conflicting, there are considerable data showing that androgen-ablation may augment an anti-tumor immune response. Enzalutamide therapy represents a more potent form of androgen suppression and may therefore be associated with previously under-appreciated immune modulatory effects. Indeed, an interesting study by Bishop et al. showed PD-L1 expression in tumor cell lines resistant to enzalutamide [21]. Those data would be consistent with the notion that responses to immunotherapy might be more common in men progressing on enzalutamide - indeed our prior case report showed an objective response to a different immunotherapy (sipuleucel-T) in precisely that setting [13].

In summary, these data provide, for the first time, evidence for meaningful clinical activity for PD-1 blockade in men with mCRPC. Although our study is limited by the small numbers of patients, the robust nature of these responses is notable, i.e. currently approved agents for mCRPC generally do not produce PSA reduction to ≤ 0.2 in the post-enzalutamide setting. Responses in liver metastases are also relatively uncommon with AR targeting drugs or cytotoxic chemotherapies. Additional work will be required to further determine the frequency, depth and duration of response in this setting; our ongoing study should provide additional data in that regard, and future studies are in the planning stages.

## MATERIALS AND METHODS

This is a single-institution, single-arm, phase II study. Male patients aged 18 years or older had histologically or cytologically confirmed adenocarcinoma of the prostate, at least one distant metastatic lesion, castrate levels of testosterone (testosterone < 50 ng/dl), Eastern Cooperative Oncology Group (ECOG) performance status of 0 or 1, and adequate organ function. They must have had a prostate specific antigen (PSA) response to enzalutamide, which was defined as a PSA decrease of ≥ 50%, and at the time of enrollment must have demonstrated progression on enzalutamide as defined by Prostate Cancer Working Group Criteria 2 and could have either PSA or radiographic progression, but not clinical progression (i.e. pain). They could not have received chemotherapy for castration resistant disease, but prior chemotherapy for castration sensitive disease was permitted. They could have received prior abiraterone or sipuleucel-T. Patients could not participate if they had known HIV, hepatitis B, hepatitis C or brain metastases. They could not have active autoimmune diseases that required systemic treatment within the past 3 months, or a documented history of clinically severe autoimmune disease that required hospitalization or significant medical intervention. Prior exposure to PD-1, PD-L1 or CTLA-4 inhibitors was not allowed. Patients with metastatic deposits amenable to biopsy were biopsied for correlative studies.

All patients were continued on enzalutamide 80-160 mg orally per day. Pembrolizumab was administered as a dose of 200 mg IV every 3 weeks for four doses. Dose delays for toxicity were permitted, but there were no dose reductions. Clinical and laboratory status was assessed every 3 weeks during active treatment and then every 6 weeks until disease progression that required a change of antineoplastic therapy.

Tumor assessments by radiographic imaging (computed tomography of the chest, abdomen, pelvis and Technitium-99 nuclear medicine bone scintagraphy) were performed every 12 weeks and serum PSA was measured every 3 weeks during active treatment with pembrolizumab and every 6 weeks in active follow up. If stable or responsive disease was observed, retreatment with pembrolizumab 200 mg IV every 3 weeks for four doses was permitted upon disease progression.

To detect antitumor activity, we chose the primary endpoint of PSA response, which was defined as a confirmed PSA decline of ≥ 50%. Secondary endpoints included objective disease response by radiographs, PSA progression free survival, and overall survival.

For those patients with a metastatic lesion that could be biopsied an image-guided biopsy was performed as previously described [22]. Immunohistochemistry was used to examine the presence and subtypes of leukocytes as well as PD-L1 expression in tumor samples. The expression of CD3 (T cells), CD8 (cytotoxic T cells), CD163 (macrophages), cytokeratin (CK; for localization of tumor cells), PD-L1, and DAPI (nuclear stain) were determined by immunohistochemistry using multispectral imaging (Vectra; PerkinElmer). Slides were deparaffinized in xylene and alcohol. For fluorescence microscopy, slides were blocked with polyclonal IgG and PeroxAbolish (Biocare Medical) before staining with rabbit anti-human CD3 (SP7) (Spring Bioscience), PD-L1 (E1L3N) (Cell Signaling), and CD8 (SP16) (Spring Bioscience) followed by the anti-rabbit secondary SuperPicTure Polymer Detection Kit (Life Technologies) and mouse anti-human CD163 (MRQ-26) (Roche) and Cytokeratin (PCK-26) (Sigma) followed by the anti-mouse secondary SuperPicTure Polymer Detection Kit (Life Technologies). The signals were amplified using TSA-Cyanine 5.5 (PerkinElmer), TSA-Cyanine 5 (PerkinElmer), TSA-Cyanine 3.5 (PerkinElmer), TSA-FITC (PerkinElmer), TSA-Coumarin (PerkinElmer). Microwave antigen retrieval in Citrate buffer (1X, pH 6.0) (Millipore) was done before each marker. Slides were mounted in Vectashield mounting medium with DAPI (Vector Laboratories) and visualized using the Vectra Microscopy imaging system (PerkinElmer). Representative regions were captured at 200X magnification using Vectra Software and were analyzed using Inform Image Analysis software (PerkinElmer).

Mismatch repair status was assessed in tumor DNA with the use or microsatellite instability analysis (Personal Genome Diagnostics) that examined was determined using five genes commonly recognized as prone to copying errors (BAT-25, BAT-26, NR-21, NR-24, MONO-27). Briefly, genomic DNA was purified from tumor and normal specimens. DNA samples were enriched for coding regions in the genome using custom DNA capture approaches. Enriched tumor and normal DNA was sequenced using massively parallel sequencing instruments. Sequence data were mapped to the reference human genome sequence and sequence alterations were determined by comparison of over 1.5 million bases of tumor and normal DNA.

The primary analysis endpoint was PSA response rate. This study was designed to ensure a 90% power (two-sided α = 0.05) to reject the null hypothesis of no response to pembrolizumab. The alternate hypothesis is a 25% response rate. The sample size to achieve that is 28. The unexpected activity noted here prompted this early report while the study continues.

This study is registered on clinicaltrials.gov (NCT02312557) and is being performed under all applicable regulatory guidelines.

## References

[1] Topalian SL, Hodi FS, Brahmer JR, Gettinger SN, Smith DC, McDermott DF, Powderly JD, Carvajal RD, Sosman JA, Atkins MB, Leming PD, Spigel DR, Antonia SJ (2012). Safety, activity, and immune correlates of anti-PD-1 antibody in cancer. N Engl J Med.

[2] Borghaei H, Paz-Ares L, Horn L, Spigel DR, Steins M, Ready NE, Chow LQ, Vokes EE, Felip E, Holgado E, Barlesi F, Kohlhaufl M, Arrieta O (2015). Nivolumab versus Docetaxel in Advanced Nonsquamous Non-Small-Cell Lung Cancer. N Engl J Med.

[3] Brahmer J, Reckamp KL, Baas P, Crino L, Eberhardt WE, Poddubskaya E, Antonia S, Pluzanski A, Vokes EE, Holgado E, Waterhouse D, Ready N, Gainor J (2015). Nivolumab versus Docetaxel in Advanced Squamous-Cell Non-Small-Cell Lung Cancer. N Engl J Med.

[4] Motzer RJ, Escudier B, McDermott DF, George S, Hammers HJ, Srinivas S, Tykodi SS, Sosman JA, Procopio G, Plimack ER, Castellano D, Choueiri TK, Gurney H (2015). Nivolumab versus Everolimus in Advanced Renal-Cell Carcinoma. N Engl J Med.

[5] Robert C, Schachter J, Long GV, Arance A, Grob JJ, Mortier L, Daud A, Carlino MS, McNeil C, Lotem M, Larkin J, Lorigan P, Neyns B (2015). Pembrolizumab versus Ipilimumab in Advanced Melanoma. N Engl J Med.

[6] Robert C, Long GV, Brady B, Dutriaux C, Maio M, Mortier L, Hassel JC, Rutkowski P, McNeil C, Kalinka-Warzocha E, Savage KJ, Hernberg MM, Lebbe C (2015). Nivolumab in previously untreated melanoma without BRAF mutation. N Engl J Med.

[7] Rosenberg JE, Hoffman-Censits J, Powles T, van der Heijden MS, Balar AV, Necchi A, Dawson N, O'Donnell PH, Balmanoukian A, Loriot Y, Srinivas S, Retz MM, Grivas P (2016). Atezolizumab in patients with locally advanced and metastatic urothelial carcinoma who have progressed following treatment with platinum-based chemotherapy: a single-arm, multicentre, phase 2 trial. Lancet.

[8] Martin AM, Nirschl TR, Nirschl CJ, Francica BJ, Kochel CM, van Bokhoven A, Meeker AK, Lucia MS, Anders RA, DeMarzo AM, Drake CG (2015). Paucity of PD-L1 expression in prostate cancer: innate and adaptive immune resistance. Prostate Cancer Prostatic Dis.

[9] Kwon ED, Drake CG, Scher HI, Fizazi K, Bossi A, van den Eertwegh AJ, Krainer M, Houede N, Santos R, Mahammedi H, Ng S, Maio M, Franke FA (2014). Ipilimumab versus placebo after radiotherapy in patients with metastatic castration-resistant prostate cancer that had progressed after docetaxel chemotherapy (CA184-043): a multicentre, randomised, double-blind, phase 3 trial. Lancet Oncol.

[10] Communication Y Per Press Release by Bristol Myers Squibb.

[11] Mercader M, Bodner BK, Moser MT, Kwon PS, Park ES, Manecke RG, Ellis TM, Wojcik EM, Yang D, Flanigan RC, Waters WB, Kast WM, Kwon ED (2001). T cell infiltration of the prostate induced by androgen withdrawal in patients with prostate cancer. Proc Natl Acad Sci U S A.

[12] Roden AC, Moser MT, Tri SD, Mercader M, Kuntz SM, Dong H, Hurwitz AA, McKean DJ, Celis E, Leibovich BC, Allison JP, Kwon ED (2004). Augmentation of T cell levels and responses induced by androgen deprivation. J Immunol.

[13] Graff JN, Drake CG, Beer TM (2013). Complete biochemical (prostate-specific antigen) response to sipuleucel-T with enzalutamide in castration-resistant prostate cancer: a case report with implications for future research. Urology.

[14] Graff JN, Puri S, Bifulco CB, Fox BA, Beer TM (2014). Sustained complete response to CTLA-4 blockade in a patient with metastatic, castration-resistant prostate cancer. Cancer Immunol Res.

[15] Drake CG, Doody AD, Mihalyo MA, Huang CT, Kelleher E, Ravi S, Hipkiss EL, Flies DB, Kennedy EP, Long M, McGary PW, Coryell L, Nelson WG (2005). Androgen ablation mitigates tolerance to a prostate/prostate cancer-restricted antigen. Cancer Cell.

[16] Ardiani A, Farsaci B, Rogers CJ, Protter A, Guo Z, King TH, Apelian D, Hodge JW (2013). Combination therapy with a second-generation androgen receptor antagonist and a metastasis vaccine improves survival in a spontaneous prostate cancer model. Clin Cancer Res.

[17] Gannon PO, Poisson AO, Delvoye N, Lapointe R, Mes-Masson AM, Saad F (2009). Characterization of the intra-prostatic immune cell infiltration in androgen-deprived prostate cancer patients. J Immunol Methods.

[18] Garon EB, Rizvi NA, Hui R, Leighl N, Balmanoukian AS, Eder JP, Patnaik A, Aggarwal C, Gubens M, Horn L, Carcereny E, Ahn MJ, Felip E (2015). Pembrolizumab for the treatment of non-small-cell lung cancer. N Engl J Med.

[19] Robinson D, Van Allen EM, Wu YM, Schultz N, Lonigro RJ, Mosquera JM, Montgomery B, Taplin ME, Pritchard CC, Attard G, Beltran H, Abida W, Bradley RK (2015). Integrative clinical genomics of advanced prostate cancer. Cell.

[20] Pritchard CC, Morrissey C, Kumar A, Zhang X, Smith C, Coleman I, Salipante SJ, Milbank J, Yu M, Grady WM, Tait JF, Corey E, Vessella RL (2014). Complex MSH2 and MSH6 mutations in hypermutated microsatellite unstable advanced prostate cancer. Nat Commun.

[21] Bishop JL, Sio A, Angeles A, Roberts ME, Azad AA, Chi KN, Zoubeidi A (2015). PD-L1 is highly expressed in Enzalutamide resistant prostate cancer. Oncotarget.

[22] Epstein JI, Amin MB, Beltran H, Lotan TL, Mosquera JM, Reuter VE, Robinson BD, Troncoso P, Rubin MA (2014). Proposed morphologic classification of prostate cancer with neuroendocrine differentiation. Am J Surg Pathol.

